# Posterior Dislocation of the Sternoclavicular Joint in a Patient With Hemophilia: A Case Report

**DOI:** 10.7759/cureus.59688

**Published:** 2024-05-05

**Authors:** Shu Somemura, Yohei Shimada, Yosuke Kano, Koh Terauchi, Hisateru Niki

**Affiliations:** 1 Department of Orthopaedic Surgery, St. Marianna University School of Medicine, Kawasaki, JPN

**Keywords:** reconstruction, sports injury, sternoclavicular joint, posterior dislocation, hemophiliac

## Abstract

Posterior sternoclavicular joint (SCJ) dislocations are rare but serious injuries. We report our experience with a patient with hemophilia who experienced posterior dislocation of the SCJ and was treated with an open repair technique.

A 17-year-old man with hemophilia had a posterior dislocation of the SCJ and the proximal clavicle was an approximation to the brachiocephalic artery. Cardiovascular surgeons and pediatricians were consulted on the day of injury. The patient underwent open reduction of the SCJ and the SCJ was stabilized with strong sutures using a tension-band technique. The patient returned to playing rugby three months after surgery.

Posterior dislocation of the SCJ has a risk of vascular injury. Although our patient required more attention because of his hemophilia, the surgery was successful through collaboration with other departments. Reconstruction of the SCJ using a tension-band technique with strong sutures was useful and allowed early return to sports.

## Introduction

Sternoclavicular joint (SCJ) dislocations account for 1% of all dislocations, and posterior dislocations are even rarer, at 5% of all SCJ dislocations [1]. Hemophilia is a genetic disease that causes abnormal bleeding, and individuals with hemophilia are at risk for bleeding, bruising, and joint damage when participating in sports [2]. However, recent advances in medicine have made hemophilia more manageable, and hemophilia guidelines now recommend participation in sports activities for health benefits [3]. In this report, we describe our experience with a patient with hemophilia who experienced a posterior dislocation of the SCJ and underwent open reduction and reconstruction.

## Case presentation

**Figure 1:**
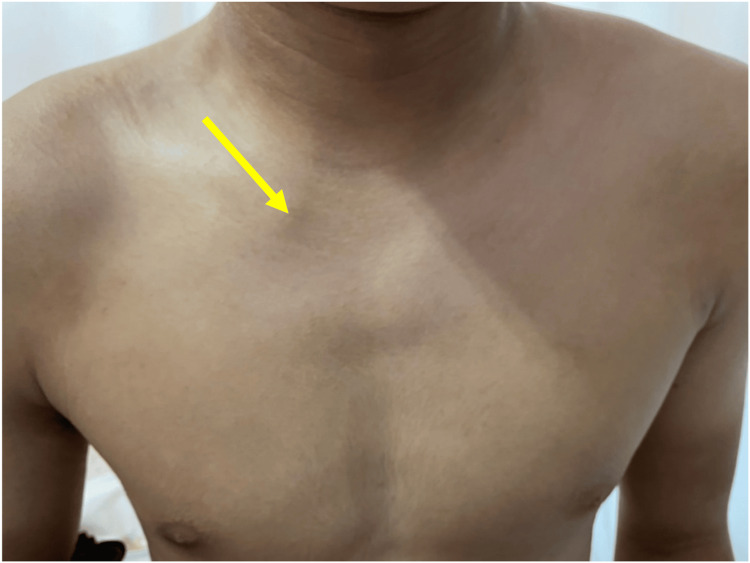
Palpable deficiency in the right SCJ (→). SCJ, sternoclavicular joint

**Figure 2:**
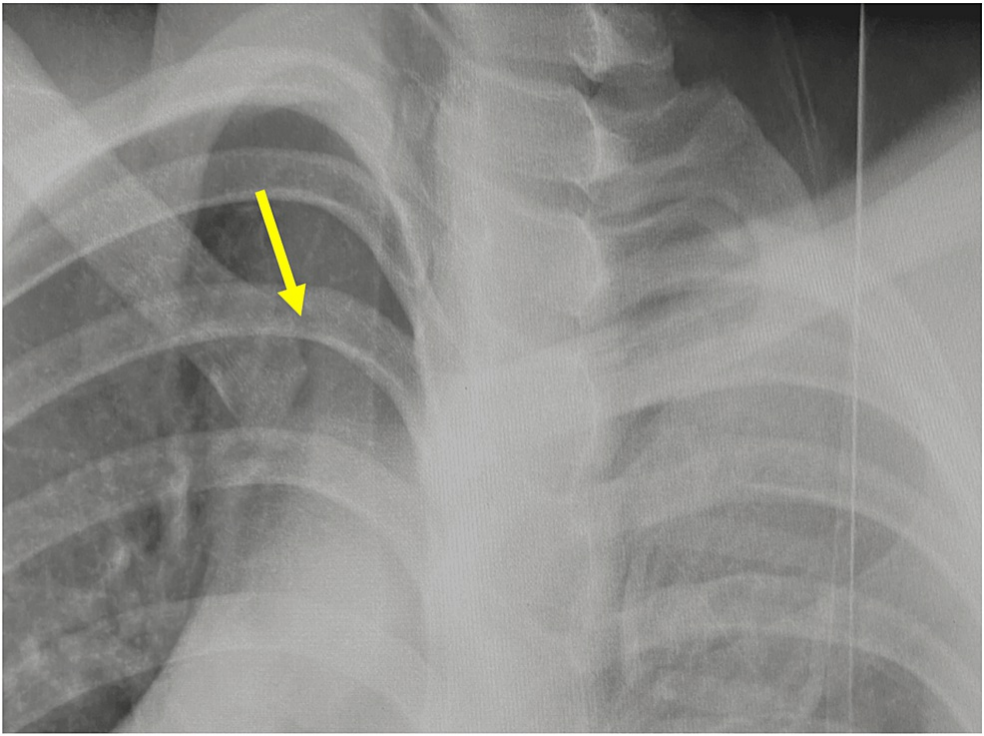
On oblique radiography, the right clavicle was slightly displaced distally (→).

**Figure 3:**
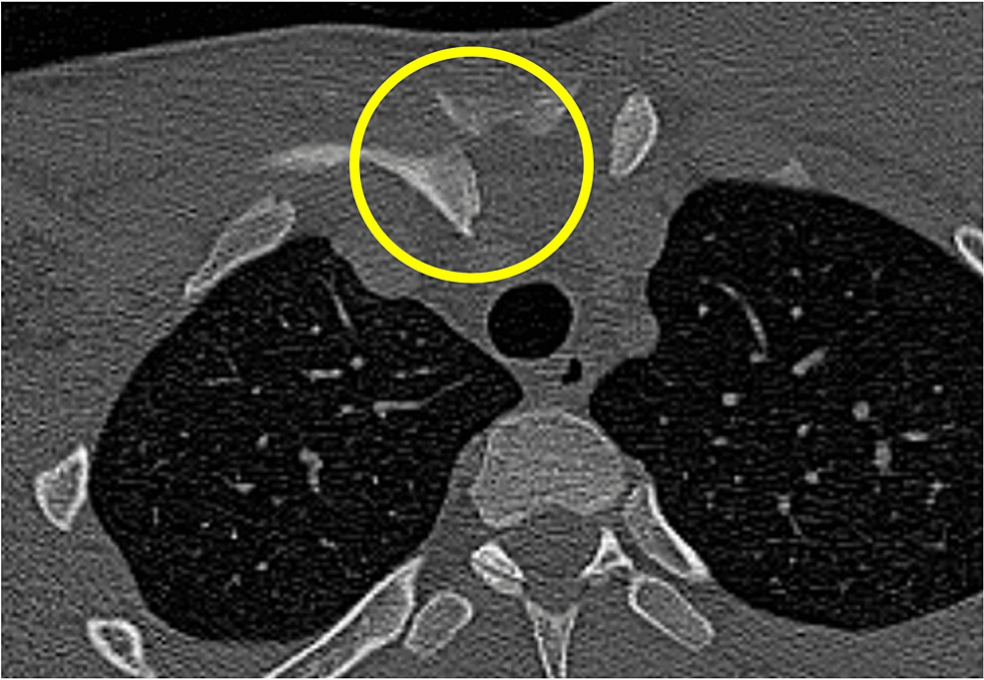
The right proximal clavicle was posteriorly dislocated without any evidence of fracture to the medial end of the clavicle.

**Figure 4:**
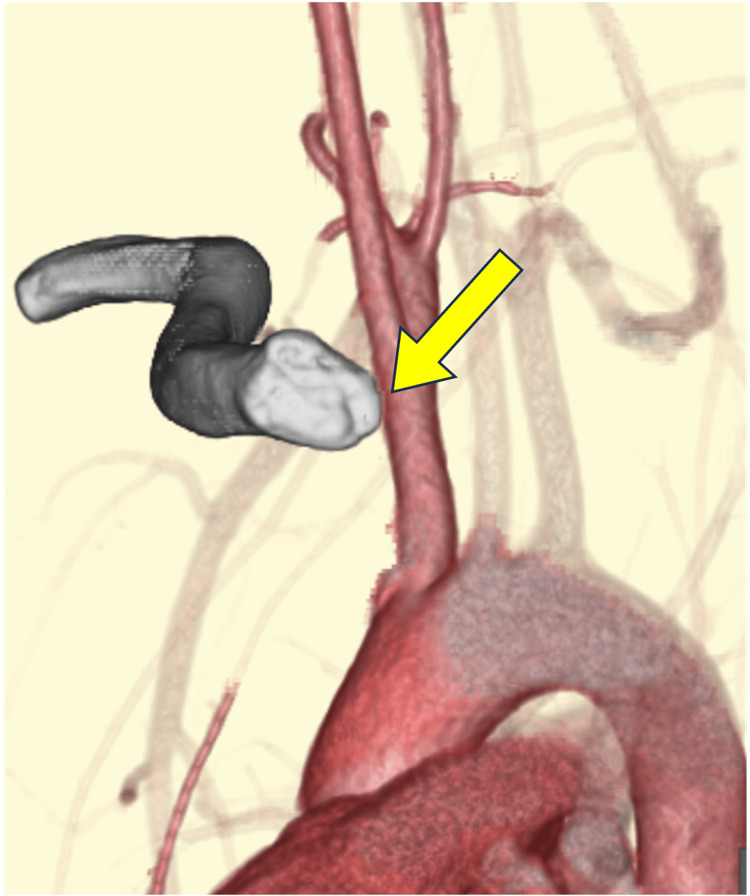
The proximal clavicle approximated the brachiocephalic artery (→).

A 17-year-old male high school senior and rugby player had been diagnosed with hemophilia B at the age of 12. His hemophilia was well controlled on regular clotting factor replacement therapy, and his pediatricians allowed him to participate in sports without restrictions. While playing rugby, he sustained an injury from a tackle that caused his right shoulder to hit the ground. On presentation, there was a palpable deficiency of the right SCJ area (Figure 1) and he reported right anterior chest pain, mild dysphagia, and mild dyspnea. On oblique radiography, the right clavicle was slightly displaced distally. Contrast-enhanced computed tomography (CT) showed that the proximal clavicle was displaced posteriorly, approximating the brachiocephalic artery and trachea (Figures 2, 3, 4). We consulted with cardiovascular surgeons and pediatricians before proceeding to the operating room for open reduction four days after the injury.

**Figure 5:**
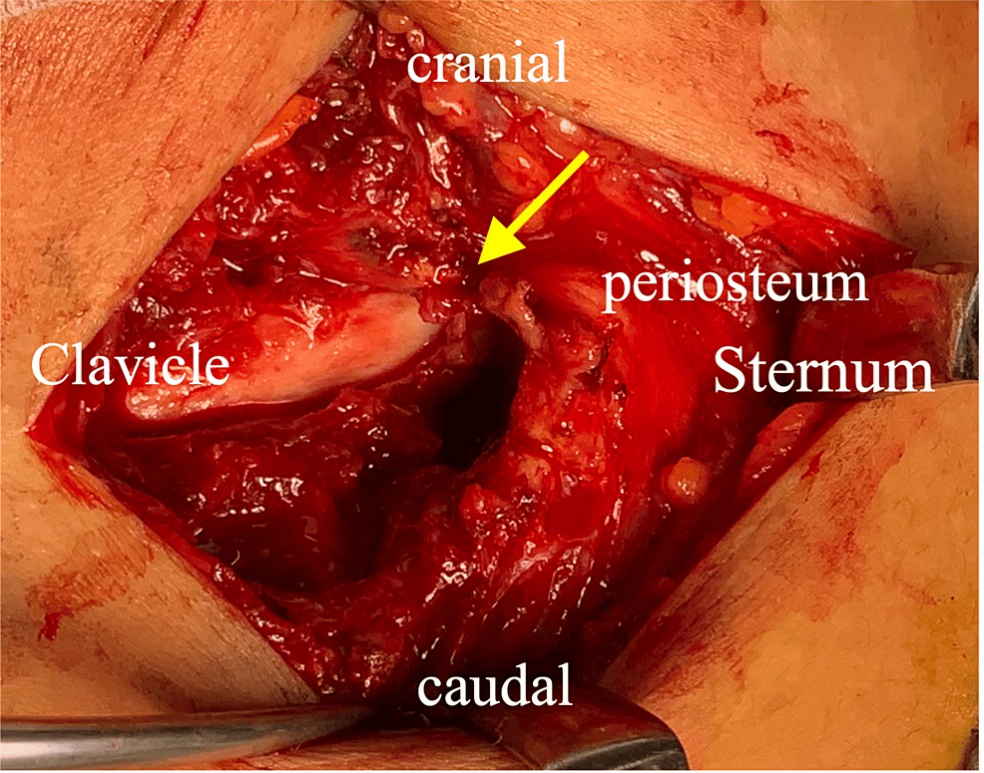
The clavicle was displaced posteriorly and was found to be interrupted by the periosteum (→).

**Figure 6:**
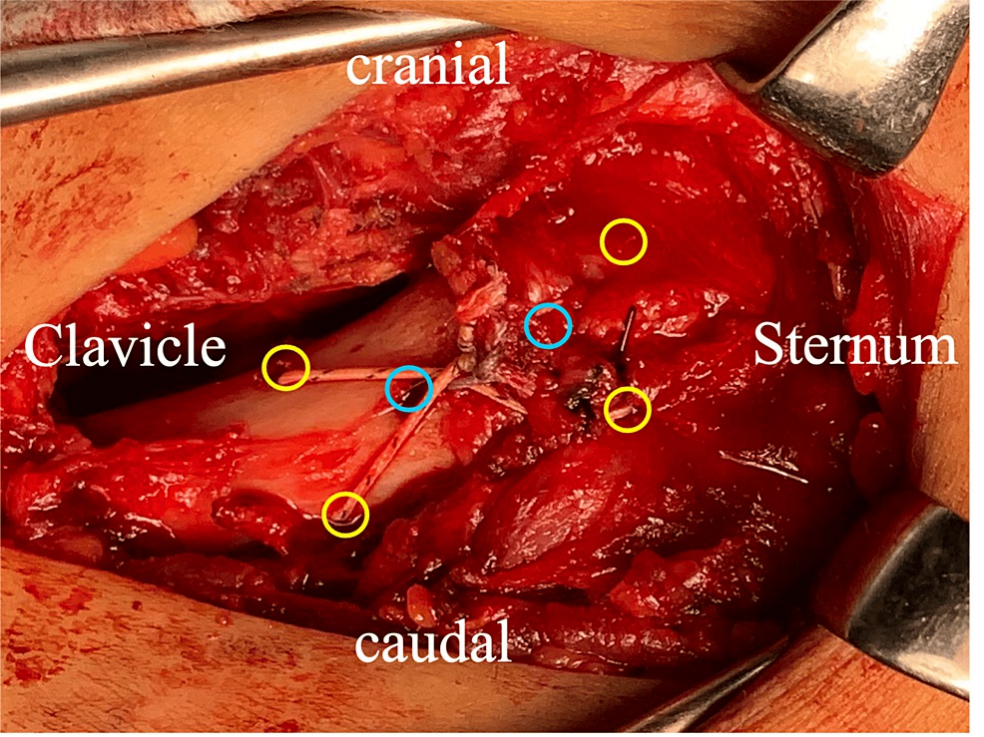
The reconstruction with a tension band technique using strong sutures (yellow circle) and additional horizontal sutures (blue circle).

During the procedure, the cardiovascular surgeon was on call; the pediatrician was responsible for replacing the patient's coagulation factor during the perioperative period. This preparation allowed the surgery to proceed with the patient experiencing blood loss comparable to that of a normal patient. During the surgery, the patient was placed in a supine position under general anesthesia, and a rolled towel was placed between the scapulae. A 6 cm incision was centered on the medial aspect of the clavicle and extended to the sternum. At this point, it was clear that the injury was a Salter-Harris type I injury that involved the junction of the growth plate and the metaphysis of the medial clavicle. The clavicle was displaced posteriorly and the periosteum was interrupted (Figure 5). An abduction-traction maneuver was attempted, but it was not possible to reduce the dislocation; dissection of the medial clavicular metaphyseal periosteum was required to reduce the dislocated fragment. We used a reduction clamp on the medial clavicular metaphysis and axial traction and extension of the shoulder to achieve reduction. Because the growth plate was still unstable, we completed the reconstruction with a tension-band technique using strong sutures. A unicortical 2.0 mm Kirschner wire bony hole was created 2 cm lateral to the medial metaphyseal surface of the clavicle and anteriorly superior and inferior to the clavicle, and a #2 jacketed polyethylene wire (FiberWire; Arthrex, Naples, FL) was passed through this hole. Similar bony holes were made in the clavicle epiphysis, and a figure-eight tension-band technique was used to secure the clavicle. Additional horizontal sutures were placed (Figure 6), and the growth plate was confirmed to be stabilized. The surgery was completed without major vascular injury. 

**Figure 7:**
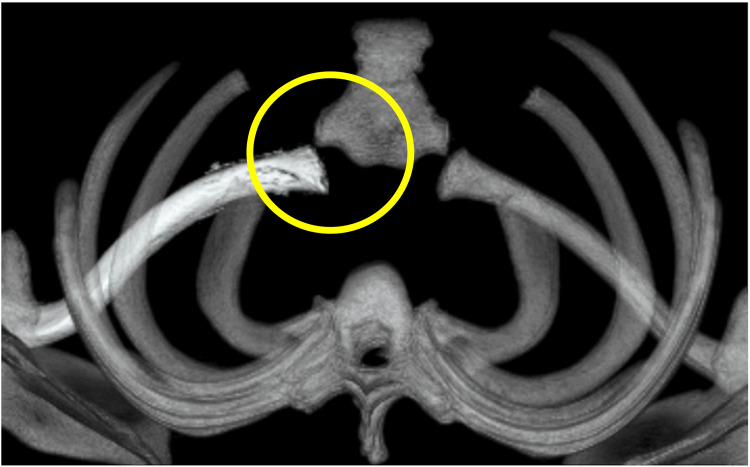
The clavicle is repaired.

The patient’s dysphagia and dyspnea improved rapidly after surgery. His right arm was immobilized with a sling for three weeks postoperatively. Then moved to gentle mobility and strengthening exercises, and concluded with sport-specific training, integrating hemophilia management throughout. At three months postoperatively, the patient achieved a perfect score on the Constant Murley Shoulder Outcome Score, indicating optimal shoulder function across pain levels, daily activities, range of motion, and strength, thereby enabling clearance to resume playing rugby. Outpatient follow-up CT was performed six months after the injury and showed satisfactory maintenance of the reduction (Figure 7). At 12 months, the patient had full, pain-free function. The patients and their families were informed that data from the research would be submitted for publication and gave their consent.

## Discussion

Posterior dislocation of the SCJ is less common than other types of dislocation because the posterior SCJ is thicker and stronger than the anterior ligament [4]. Posterior dislocation can be caused by either a direct force on the proximal clavicle, from anterior to posterior, or an indirect twisting force resulting from the clavicle pivoting on the first rib as the shoulder girdle is pushed backward [5]. Our patient's right shoulder hit the ground due to a rugby tackle, and the indirect force is thought to have caused the posterior dislocation of the SCJ. 

Due to the low incidence of this injury, many surgeons have limited experience with its management. The proximal clavicle is close to vital organs such as the lungs, esophagus, and great vessels, making posterior dislocation of the SCJ a potentially life-threatening injury [6,7]. Worman et al. reported esophageal and tracheal macrovascular injury in 16 of 60 patients with posterior dislocation of the SCJ, with two deaths resulting from these injuries [8]. Our patient had dysphagia and dyspnea caused by esophageal and tracheal compression, and the proximal end of the clavicle was impinging on the brachiocephalic artery, placing the patient at high risk for vascular injury. In addition, the patient had hemophilia, which further increased the surgical complexity. Hemophilia is a congenital bleeding disorder with X-linked recessive inheritance. Bleeding symptoms usually begin in childhood. Surgery in patients with hemophilia carries a risk of excessive bleeding and requires replacement of clotting factors. Although our patient had mild hemophilia B and his disease was well controlled with regular factor replacement, additional perioperative coagulation factor replacement allowed the surgery to proceed without complications. 

As demonstrated by this patient, it is difficult to distinguish between a true posterior dislocation and a posterior displacement of an epiphyseal disruption of the medial clavicle on conventional radiographs or even CT. The true nature of the injury can only be verified during open reduction, or retrospectively when new bone formation and bone remodeling are seen on follow-up CT. For this reason, many reports do not make a clear distinction between these two injuries, and they are both generally reported and treated as posterior dislocations of the SCJ [9]. 

The optimal method of stabilizing the SCJ has not been established; various methods including Kirschner wires, plate and screw fixations, strong sutures, and autogenous tendon grafts have been reported [10]. The SCJ has a vertical axis from superior and medial to inferior and lateral and an anteroposterior axis from posterior and medial to anterior and lateral, which allows movement in all directions as well as approximately 30 degrees of posterior rotation [4]. Although some authors have previously recommended percutaneous Kirschner wire fixation, a type of rigid fixation, it is certainly not recommended today due to the possibility of wire migration with penetration of major vessels [11]. Fixation of the SCJ with plates or screws also results in rigid fixation, risking implant failure and possibly requiring removal [12]. Autogenous tendon grafting for ligament reconstruction is another option, but there may be problems with tendon harvesting such as elongation or atrophy [13]. Aydin et al. describe the tension-band technique using strong sutures, which is a less invasive and semi-rigid fixation, making it useful for this scenario [14]. We used a similar method and found it to be particularly safe and effective for patients with a high risk of bleeding, as in the current case. Suture fixation is sufficient for this injury because it is relatively stable after reduction [15]. We used a similar method and found it to be safe and effective. Our patient was able to return to playing rugby three months after surgery.

## Conclusions

We encountered a rare traumatic posterior dislocation of the SCJ in a patient with hemophilia. The patient was at risk for major vascular injury, but by collaborating with cardiovascular surgeons and pediatricians, the surgery was performed without complications. The SCJ was reconstructed using the tension-band technique with strong sutures, which provided firm fixation and allowed the patient to return to rugby in the early postoperative period.

## References

[1] Ciftdemir M, Copuroglu C, Ozcan M (2011). Posterior dislocation of the sternoclavicular joint. Hippokratia.

[2] Seuser A, Boehm P, Kurme A, Schumpe G, Kurnik K (2007). Orthopaedic issues in sports for persons with haemophilia. Haemophilia.

[3] Srivastava A, Brewer AK, Mauser-Bunschoten EP (2013). Guidelines for the management of hemophilia. Haemophilia.

[4] Spencer EE, Kuhn JE, Huston LJ, Carpenter JE, Hughes RE (2002). Ligamentous restraints to anterior and posterior translation of the sternoclavicular joint. J Shoulder Elbow Surg.

[5] Sewell MD, Al-Hadithy N, Le Leu A, Lambert SM (2013). Instability of the sternoclavicular joint: current concepts in classification, treatment and outcomes. Bone Joint J.

[6] Marker LB, Klareskov B (1996). Posterior sternoclavicular dislocation: an American football injury. Br J Sports Med.

[7] Lee JT, Nasreddine AY, Black EM, Bae DS, Kocher MS (2014). Posterior sternoclavicular joint injuries in skeletally immature patients. J Pediatr Orthop.

[8] Worman LW, Leagus C (1967). Intrathoracic injury following retrosternal dislocation of the clavicle. J Trauma.

[9] Garg S, Alshameeri ZA, Wallace WA (2012). Posterior sternoclavicular joint dislocation in a child: a case report with review of literature. J Shoulder Elbow Surg.

[10] Glass ER, Thompson JD, Cole PA, Gause TM 2nd, Altman GT (2011). Treatment of sternoclavicular joint dislocations: a systematic review of 251 dislocations in 24 case series. J Trauma.

[11] Van Le M, Swap C (2013). Image diagnosis: a 16 year old with chest pain after blunt trauma. Perm J.

[12] Quayle JM, Arnander MW, Pennington RG, Rosell LP (2014). Artificial ligament reconstruction of sternoclavicular joint instability: report of a novel surgical technique with early results. Tech Hand Up Extrem Surg.

[13] Castropil W, Ramadan LB, Bitar AC, Schor B, de Oliveira D'Elia C (2008). Sternoclavicular dislocation—reconstruction with semitendinosus tendon autograft: a case report. Knee Surg Sports Traumatol Arthrosc.

[14] Aydın E, Dülgeroğlu TC, Ateş A, Metineren H (2015). Repair of unstable posterior sternoclavicular dislocation using nonabsorbable tape suture and tension band technique: a case report with good results. Case Rep Orthop.

[15] Swarup I, Hughes MS, Cazzulino A, Spiegel DA, Shah AS (2020). Open reduction and suture fixation of acute sternoclavicular fracture-dislocations in children. JBJS Essent Surg Tech.

